# A megastudy of behavioral interventions to promote frequent HIV testing among adults at high risk of HIV infection in Kenya and Uganda: study protocol for a randomized controlled trial

**DOI:** 10.1186/s13063-026-09783-4

**Published:** 2026-05-21

**Authors:** Kara Marson, Jane Kabami, Alison M. Buttenheim, Daniel Bennett, Maya L. Petersen, Diane V. Havlir, Moses R. Kamya, James Ayieko, Harsha Thirumurthy, Gabriel Chamie

**Affiliations:** 1 Division of HIV, Infectious Diseases, and Global Medicine, University of California San Francisco, San Francisco, CA USA; 2 Infectious Diseases Research Collaboration, Kampala, Uganda; 3 Department of Family and Community Health, University of Pennsylvania, Philadelphia, PA USA; 4 Center for Economic and Social Research, University of Southern California, Los Angeles, USA; 5 Division of Biostatistics, University of California, Berkeley, CA USA; 6 School of Medicine, Makerere University College of Health Sciences, Kampala, Uganda; 7 Kenya Medical Research Institute, Nairobi, Kenya; 8 Department of Medical Ethics and Health Policy, University of Pennsylvania, Philadelphia, PA USA

**Keywords:** HIV prevention, HIV retesting, Behavioral science, Megastudies, Randomized controlled trial

## Abstract

**Background:**

Frequent HIV testing among people at high risk of HIV infection is critical for early diagnosis and prompt antiretroviral therapy (ART) initiation for those who test HIV positive, as well as provision of effective biomedical HIV prevention modalities like pre-exposure prophylaxis (PrEP) for those who test negative. Despite World Health Organization (WHO) guidelines recommending retesting for HIV every 3–6 months for adults at increased risk**,** regular retesting is infrequent in sub-Saharan Africa. Large-scale, multi-arm field experiments (“megastudies”) can evaluate many different behavioral interventions among the same population and at the same time rapidly identify behavioral solutions and overcome the challenge of comparing results across standalone trials. We describe the protocol for a megastudy that will simultaneously test multiple low-cost behavioral interventions to increase HIV retesting among adults at high risk of HIV infection in rural Kenya and Uganda.

**Methods:**

We will conduct a 12-arm randomized trial of light-touch (“nudge”) behavioral interventions among 30,000–40,000 HIV-uninfected adults aged ≥ 15 years in western Kenya and southwestern Uganda who self-report increased risk of HIV infection and have access to a mobile phone. Following a baseline HIV-negative test at a high-volume HIV testing center, participants will be individually randomized in parallel to one of 12 arms, including 10 different intervention arms comprising low-cost messages that serve as nudges for retesting, an intervention arm with a financial incentive for retesting, or a control arm with standard of care post-test counseling services. The primary outcome will be HIV retesting at any study venue 3–6 months after the initial baseline HIV test. We will also measure HIV-positivity, linkage to care and ART initiation, and PrEP referral acceptance at follow-up.

**Discussion:**

This study will provide new information on low-cost interventions to promote HIV retesting for adults at elevated risk of infection in East Africa. The methods and results of this study will provide valuable insights for those engaged in HIV service delivery and influence how researchers and programs design and test low-cost, scalable interventions to promote health behaviors.

**Trial registration:**

ClinicalTrials.gov NCT06971367. Registered on May 6, 2025, https://clinicaltrials.gov/study/NCT06971367

## Introduction

### Background and rationale

Frequent HIV testing among people at increased HIV risk is crucial for epidemic control. For those who have recently become infected, frequent testing is critical for early diagnosis and prompt antiretroviral therapy (ART) initiation, thus reducing morbidity and eliminating HIV transmission risk [1–3]. For those who are HIV-negative but remain at higher risk of HIV infection, retesting enables provision of effective biomedical HIV prevention modalities like pre-exposure prophylaxis (PrEP). World Health Organization (WHO) guidelines recommend retesting every 3–6 months for adults at increased HIV risk [4].

Despite WHO guidelines, regular retesting is infrequent in sub-Saharan Africa (SSA) and results in delayed HIV diagnosis following infection. Several surveys show that many adults have not had an HIV test in the prior year [5–7] and that the median time to HIV diagnosis following infection remains > 2.5 years even as the proportion of adults who have ever tested for HIV increases [8]. Global estimates of CD4^+^ cell count at HIV diagnosis and ART initiation are unacceptably low, with ~50% of people starting ART globally at CD4^+^ counts < 350 cells/μL in studies from the 2010s [9, 10]. Late diagnosis results in missed opportunities for HIV treatment as prevention along with avoidable morbidity and mortality [1–3].

Behavioral barriers to HIV retesting are distinct from barriers to one-time testing. In a study among adults in southwestern Uganda at increased risk of HIV recruited from bars, sites of commercial sex work, and transport hubs, we found that although most adults (> 80%) reported ever testing for HIV, few (18%) reported regular retesting for HIV every 3 months in accordance with Uganda guidelines [11]. Similar findings have been reported elsewhere in SSA [12–14]. The factors driving low retesting rates include low perceived HIV risk based on a prior HIV-negative test result [15], inaccurate mental models of HIV infection (e.g., the belief that retesting is unnecessary if a person continues to feel healthy [16, 17]), and decreased salience of or focus on prevention over time (i.e., prevention fatigue) [18, 19].

In a wide range of settings, behavioral science research has demonstrated the potential for addressing barriers to various health behaviors with low-cost interventions, including “nudges” that encourage specific actions without restricting choices [20, 21]. These include messages that frame health information in salient ways and leverage behavioral economic patterns (e.g., by leveraging status quo bias through the use of default appointments) [22]. Such interventions have shown promise in promoting many health behaviors, but they have seldom been applied to retesting. Moreover, since individual behavioral interventions to increase re-testing have been evaluated in different settings, populations, and time periods, it is difficult to make like-to-like comparisons across retesting interventions and select the one most likely to boost retesting rates for implementation.

Large-scale, multi-arm field experiments (sometimes referred to as “megastudies”) that feature individual randomization and evaluate many different behavioral interventions among the same population and at the same time can rapidly identify behavioral solutions and overcome the challenge of comparing results across standalone trials. In recent megastudies, researchers have sought to identify solutions for public health priorities in the USA like exercise and flu vaccination uptake in large populations (from 30,000 to 3.1 million persons) [23–26]. Such trials make it possible to test the efficacy of *many*interventions in the same population for the same outcome [27]. This approach also increases the speed and reduces the cost with which we learn about each intervention, thus accelerating scientific discovery.

There are few evidence-based interventions to promote retesting for HIV among high-risk persons. A recent systematic review of interventions to promote retesting found ten studies (only three of which were in SSA) and reported that text message reminders were most effective [28]. In a recent trial we conducted in Uganda, small financial incentives significantly increased retesting 3 and 6 months after a negative test among adults at increased risk of HIV [29]. In the present trial, we will leverage the megastudy approach to adapt, test, and compare multiple low-cost behavioral interventions to increase HIV retesting among adults in rural Kenya and Uganda.

## Methods

### Objectives

The Innovative Behavioral Intervention Strategies (IBIS) megastudy is designed to determine the effectiveness of multiple behavioral interventions to promote HIV retesting among adults at increased risk of HIV infection. The study will also estimate heterogeneous treatment effects to determine which interventions are most effective for priority sub-populations such as youth populations and individuals with multiple sex partners.

### Trial design

We will conduct a 12-arm randomized trial of multiple behavioral economics interventions compared to a standard of care control arm. Participants will be individually randomized in parallel to one of 10 different intervention arms that offer low-cost messages that serve as nudges for retesting, an intervention arm with a financial incentive for retesting, or a control arm with standard of care HIV post-test counseling services. The financial incentive arm is intended to provide a benchmark against which the effectiveness of the other behavioral nudges can be evaluated.

### Methods: participants, interventions, and outcomes

#### Study setting

This study will take place in Homa Bay and Migori Counties in western Kenya and Bushenyi and Ibanda Districts in southwestern Uganda. Recruitment and study activities will occur in eight hospitals and clinics that provide HIV testing services (HTS) as well as HIV prevention (e.g., pre-exposure prophylaxis (PrEP)) and antiretroviral therapy (ART) services. Specific hospitals and clinics were selected based on the number of adults who accessed HTS in recent years, thereby leveraging existing high-volume testing service infrastructures to provide a sufficient population base for the study.

#### Eligibility criteria

Individuals who undergo HIV testing at study-associated health facilities will be screened for the following eligibility criteria: age ≥ 15 years, negative HIV antibody test result at time of enrollment, no intent to migrate out of the community in the next 3 months, daily access to a mobile phone, and self-reported increased risk of HIV infection (defined by national (Kenya or Uganda) MoH criteria [30, 31]). Access to a mobile phone is a requirement because some of the interventions will be reinforced by SMS messages in the 3 months after enrollment. 

#### Ethics and consent

Because of the minimal risk associated with the behavioral interventions, we will obtain verbal informed consent from participants prior to enrollment in the study. All informed consent procedures will be conducted by trained research assistants and HTS counselors in Kiswahili or Dholuo in Kenya, Runyankole or Luganda in Uganda, or English (both countries), based on the participant’s preference. Consent will be obtained by trained research assistants and Ministry of Health (MoH) counselors. The study protocol, informed consent and procedures have been approved by the University of California, San Francisco Institutional Review Board (UCSF IRB), the Makerere University School of Medicine Research and Ethics Committee (SOMREC), and the Kenya Medical Research Institute Scientific and Ethics Review Unit (KEMRI-SERU). If there are any protocol amendments affecting study participation or informed consent, those will be shared with enrolled participants at their follow-up visit.

#### Random allocation

After eligibility is determined and the baseline questionnaire is completed, enrolled participants will be randomized to one of the 12 study arms using parallel group randomization, stratified by sex and by study site, using a computer-generated sequence with permuted blocks of size 36. Study arm allocation will be revealed to participants and research assistants (RA) on the study tablet, which will display a digital rotating wheel. Participants may choose when to stop the wheel’s rotation by clicking or requesting the RA to click a “stop” button, which will then reveal the randomization study arm assignment within the relevant stratum. The data analyst generated these random allocation sequences in advance, and RAs who assign participants to the interventions will not have access to the random allocation sequence.

#### Interventions

##### Intervention selection and adaptation

Based on our prior work evaluating interventions to promote HIV retesting [11, 29, 32], as well as a literature review of low-cost behavioral economic nudges, we identified eight initial candidate interventions designed to address the psychological underpinnings of barriers to HIV retesting. We also invited 36 researchers with expertise in behavioral economics, HIV testing, and HIV prevention in East Africa to propose additional behavioral interventions for consideration. All suggested interventions were reviewed by study investigators from the United States, Kenya, and Uganda for the HIV retesting barrier being addressed, the behavioral economics underpinning theory, prior evidence of effectiveness for other health behaviors, and feasibility for the IBIS study. Investigators individually reviewed and scored each submission and then met as a group to select which intervention ideas would proceed to local adaptation. From 23 suggested intervention ideas, we selected 11 interventions for adaptation. Recognizing that many promising interventions often fail because of inadequate adaptation to local context, we undertook a two-part adaptation process for candidate interventions prior to implementing them in the study: participatory prototyping workshops in both Kenya and Uganda and a pilot trial of the updated interventions at study sites.

We recruited two groups of individuals for the participatory prototyping workshops: (1) community members aged ≥ 18 years, self-reported being HIV-negative, and self-reported at least one of the following HIV risk factors in the previous 3 months: having had more than one sexual partner, having a known HIV-positive partner, having been diagnosed with a sexually transmitted infection (STI), and/or having paid or received gifts or money in exchange for sex; (2) health care workers (HCWs) such as clinical officers, nurses, or doctors who deliver HTS at Ministry of Health (MoH) clinics. Participants provided written consent, including consent to be audio recorded, prior to the start of the workshops.

We engaged workshop participants in one full day of intervention prototyping activities, conducted in the local language by facilitators from the Busara Center, a nonprofit organization based in Nairobi, Kenya with experience in conducting behavioral science and behavioral design research in low and middle-income countries. We divided participants into groups (4–8 participants each), each of which reviewed 2–4 prototype interventions and provided structured feedback on the appropriateness and acceptability of the content, language, and framing for intervention baseline videos and SMS follow-up messages. The Busara team recorded, transcribed, and coded the user experience discussions and distilled feedback on the interventions into a report for the study team.

Following workshop completion, we implemented a small-scale pilot study in each of the eight study communities to gain additional insights on intervention acceptability and delivery and further de-risk our trial. All participants provided written consent prior to enrollment in the pilot trial. Eligibility criteria and study procedures for the pilot study matched those of the study, listed below. We used participant feedback from the workshops and pilot study to revise and finalize 10 messaging intervention scripts for use in the study.

##### Megastudy interventions

**Fig. 1:**
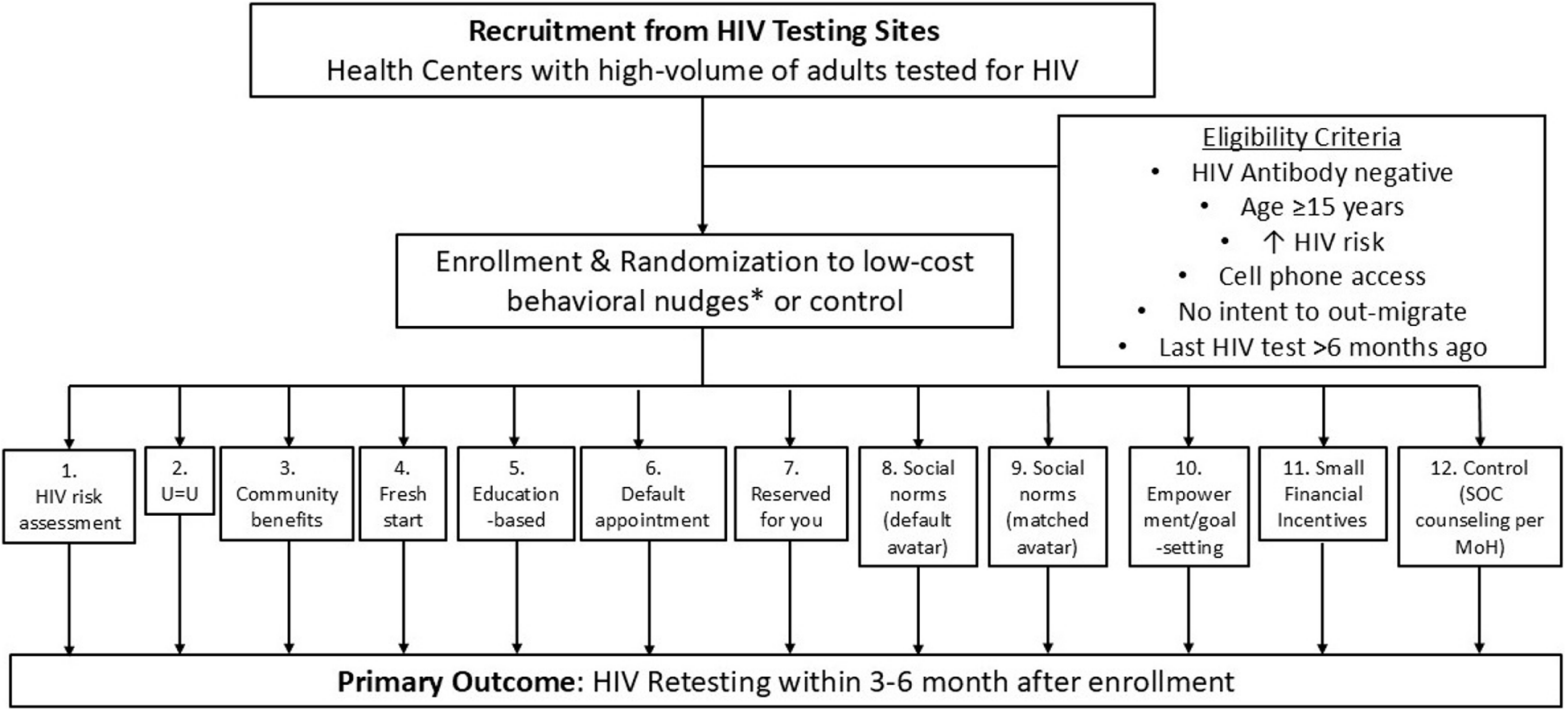
Schematic for the IBIS megastudy of interventions to promote HIV retesting among adults at increased risk of HIV infection in western Kenya and southwestern Uganda

The study will have 11 different intervention arms, of which 10 are low-cost messaging interventions and one is a small financial incentive intervention (see Table 1), and a standard care control arm. At the baseline visit, immediately after randomization, all participants assigned to one of the 10 messaging intervention arms will be shown a brief (1–2 min) avatar video message encouraging them to return for a repeat HIV test in 3 to 6 months (see Fig. 1).

**Table 1:** Megastudy interventions

Intervention	Underlying barrier and intervention rationale	Intervention content
Risk assessment	• Barrier: Low perceived HIV risk (particularly after an HIV-negative test) can be a barrier to retesting • Rationale: Prompting a self-evaluation of HIV risk can make specific risk factors more salient to participants	Brief video message about risk assessment at baseline; 2 SMS messages at 8 and 11 weeks
U = U messaging	• Barrier: Inaccurate mental models such as beliefs that one only needs an HIV test if they have symptoms, or that HIV treatment does not reduce risk of HIV transmission, can be barriers to HIV retesting • Rationale: Updating mental models with new information on asymptomatic HIV infection and modern ART (“Undetectable = Untransmittable” U = U) [33]	Brief video message about U = U messaging at baseline; 2 SMS messages at 8 and 11 weeks
Community benefits	• Barrier: Decreased salience of preventive behaviors over time can be a barrier to retesting • Rationale: Messaging that emphasizes benefits of HIV testing to one’s community can leverage altruism and thereby increase salience of engaging in preventive behaviors like retesting	Brief video message about community benefits at baseline; 2 SMS messages at 8 and 11 weeks
Fresh start effect	• Barrier: Decreased salience of preventive behaviors over time can be a barrier to HIV retesting • Rationale: “Fresh start” effect can be used to motivate health behaviors at a specific time (such as the time of a baseline, negative HIV test) [34]	Brief video message about fresh start at baseline; 2 SMS messages at 8 and 11 weeks
Standard health education about HIV	• Barrier: Lack of knowledge about basic HIV fact or belief in myths [35] • Rationale: Providing correct information about HIV is a standard public health approach and may increase understanding of the benefits of HIV retesting	Brief video message about basic HIV facts at baseline; 2 SMS messages at 8 and 11 weeks
Default appointment	• Barrier: Decreased salience of preventive behaviors over time • Rationale: Setting a default appointment time for retesting can leverage status quo bias [36] and overcome decreased salience of HIV retesting over time	Brief video message with a default appointment date at baseline; 2 SMS messages at 8 and 11 weeks
Reserved for you	• Barrier: Competing priorities can be a barrier to HIV retesting • Rationale: A message indicating that the HIV test is “reserved for you” can leverage scarcity, implied exclusivity, and loss aversion to counter this barrier [37]	Brief video message about reserved for you test kit at baseline; 2 SMS messages at 8 and 11 weeks
Social norms (default avatar)	• Barrier: Internalized or anticipated stigma can be a barrier to HIV retesting • Rationale: Messages that frame HIV testing as a prevailing social norm [38] may reduce internalized or anticipated stigma	Brief video message about social norms at baseline with default avatar (male, ~30 years); 2 SMS messages at 8 and 11 weeks
Social norms (matched avatar)	• Barrier: Internalized or anticipated stigma can be a barrier to HIV retesting • Rationale: Messages that come from a peer may be more persuasive than those delivered by someone outside of one’s peer group and framing HIV retesting as a prevailing social norm may reduce internalized or anticipated stigma	Brief video message about social norms at baseline with avatar matched to participant’s sex and age group; 2 SMS messages at 8 and 11 weeks
Empowerment/goal-setting	• Barrier: Decreased salience of preventive behaviors over time and competing priorities can be barriers to HIV retesting • Rationale: Engaging participants in goal-setting may make retesting more salient and overcome self-control problems [39]	Brief video message about empowerment and goal-setting at baseline; 2 SMS messages at 8 and 11 weeks
Financial incentive	• Barrier: The real and perceived costs of HIV testing, including cost of transportation and time taken can be barriers to retesting • Rationale: Financial incentives can offset the costs associated with retesting and also serve as an immediate benefit to some participants	Counselor tells participant at baseline that they will receive a small financial incentive (< USD $5) if they return for retesting in 3–6 months. Disbursement of financial incentive at 3–6 months retesting visit
SOC (control)		SOC MoH post-test counseling procedures

The specific message content and framing will differ based on the participant’s randomization assignment. For example, participants who have been assigned to the “HIV risk assessment” study arm will be shown a video that asks them to reflect on several specific HIV risk factors and whether they have recently engaged in any of those activities, before prompting them to return for another test. Nine of the 10 messaging arms will have the same default avatar face for consistency. Based on feedback from the local adaptation phase, a male avatar of approximately 30 years of age was selected as the default. The “social norms (matched avatar)” study arm will use an avatar face that is matched to the participant’s age (≤ 30 years/> 30 years) and sex (male/female); the message content will be the same as that for the “social norms (default avatar)” study arm.

Participants who have been assigned to one of the 10 messaging arms will also receive brief SMS follow-up messages at 8 weeks and 11 weeks after their baseline visit, reminding them to return for HIV retesting in the 3–6-month window post-enrollment. The content of these SMS messages will also be aligned with the core content categories to which participants are assigned.

Participants who are assigned to the financial incentive study arm will be told by their HIV test counselor at baseline that they will receive a small financial incentive (< US$5) if they return for another HIV test within 3 to 6 months; they will not be shown any video messaging or receive follow-up SMS reminders. Participants in all study arms, including the control, will receive standard of care HIV post-testing counseling from HIV test counselors.

#### Participant timeline and procedures

Immediately following enrollment, participants will complete a brief baseline survey including questions on demographics and recent HIV risk factors. Following the baseline survey, they will be randomized to one of the 12 study arms and, if assigned to one of the 10 messaging intervention arms, they will be shown the associated baseline video message. The study software will be programmed to automatically show only the video message for the study arm to which the participant has been randomized, to reduce possible human error. All participants will be reminded to return for another HIV test 3 to 6 months after the baseline visit. Participants who return for a repeat HIV test at follow-up will receive standard HTS through the hospital or clinic and will complete a brief follow-up study survey to assess HIV risk factors since baseline enrollment. The study team will record HIV test results as well. Participants who have been assigned to the incentive arm and return for retesting at follow-up will receive a small financial incentive (up to $5 USD) at the time of retesting. The schedule of study enrollment, interventions, and assessments is detailed in Table 2. Participants may seek and receive any concomitant care during their participation in this trial.

**Table 2:** SPIRIT figure: schedule of enrollment, allocation, interventions, and assessments in the IBIS megastudy

	Enrollment	Post-allocation		Close-out	Post-close out
Timepoint	Baseline	*t* + 8 weeks	*t* + 11 weeks	*t* + 12 weeks–*t* + 24 weeks	*t* + 25 weeks–*t* + 48 weeks
Enrollment:					
HIV testing*	X			X	
Eligibility screen	X				
Verbal consent	X				
Allocation	X				
Interventions:					
[Control arm]	X				
[Incentive arm]	X			X	
[Messaging arms, *n* = 10]	X	X	X		
Assessments:					
Questionnaire	X			X	
Post-follow up survey for non retesters**					X

^*^ HIV testing is conducted per Ministry of Health (MoH) standard of care and is not a study procedure; HIV test results will be documented on study case report forms (CRFs)

^**^ We will reach out to a small sample of participants who did not return for HIV retesting to see if they tested elsewhere and, if not, to ask about their reasons for not retesting

#### Outcomes

The primary outcome will be HIV retesting at any study venue 3–6 months after the initial baseline HIV test. We will use either participant demographic information (such as name, phone number, birthdate) and/or national identification number or electronic medical record number (if available) at baseline and time of retesting to verify participant identity (using procedures we have used in prior studies [5, 40]) and measure HIV retesting.

The secondary outcome will be PrEP referral acceptance (i.e., willingness to be referred to the PrEP clinic) among participants who retest HIV-negative and are not taking PrEP at time of retesting. We will measure acceptance of referral to PrEP services at time of retesting. Other outcomes will include the proportion of persons who test HIV positive (i.e., seroconvert) among participants who retest for HIV, as well as linkage to care and ART-initiation among those who seroconvert.

### Sample size

We made the following assumptions in determining the sample size and minimum detectable effect sizes. With 64,000 adults (≥ 15 years) testing for HIV in 2023 at the eight study HIV testing service sites and a reported HIV positivity of ~ 2%, we estimate there are about 62,700 HIV-negative adults tested per year in these study communities. We estimate that about 50% of adults (about 31,000/year) accessing HIV testing at HIV clinic sites will be classified as being at increased risk of HIV infection based on their self-reported answers to risk screening questions and thus will require quarterly HIV retesting. Based on our prior HIV retesting studies in Uganda, we anticipate at least 90% of eligible adults will agree to participate in the megatrial [29, 32]. With enrollment over 18–24 months, we anticipate enrolling 30,000–40,000 participants in the trial, with a range of 2500–3333 participants per arm in a 12-arm trial.

Based on our previous retesting study in Uganda, we assumed that 38% of the control group will retest for HIV within 6 months [29]). With a sample size of at least 3000 participants/arm, we will have 80% power to detect a 4.7 percentage-point difference in HIV testing for any of the intervention groups compared to the control group. These power calculations account for multiple hypothesis testing by using a Bonferroni correction and setting the significance level to 0.00455 (i.e., 0.05/11 comparisons between interventions and control). This is a very conservative approach; however, since we will have more statistical power in our analyses by using the Benjamini and Hochberg Procedure to control the false discovery rate [41] and covariate adjustment will also improve efficiency. Thus, in practice we expect to be able to detect differences between each intervention group and the control group that are smaller than 4.7%.

### Blinding and limitation of risk of bias

Given the nature of the study and intervention procedures, blinding is not possible for study staff nor participants. The principal investigators, co-investigators, and statistician will remain blinded to the study arm allocation of participants through the course of fieldwork and primary outcome analysis.

### Data collection and management

Clinic and study staff will enter data directly onto encrypted, password-protected study tablets at the eight participating study clinics. We have developed standard operating procedures for data collection, management, and quality assurance that will be implemented systematically across all study sites. We designed electronic data collection forms to provide clear instructions and to have automated systems to ensure correct study procedures. For example, local RAs will not be able to access the baseline survey until an eligible screening survey and informed consent have been documented. The electronic data forms also have real-time range checks and skip patterns driven by internal logic to minimize missing or incorrect data. To ensure data integrity, regular quality assurance measures will be performed by data officers in Kenya, Uganda, and the USA to assess for missing, incomplete, or invalid data fields. Study tablet data will be synced and backed up centrally to secure servers in Kenya and Uganda daily and uploaded to secure servers at the University of California, San Francisco (UCSF).

### Statistical analysis

For the primary outcome of HIV retesting, we will estimate a logistic regression model to determine the effect of each behavioral intervention. The main predictors in our regression model will be indicator variables for each intervention group, with the reference being the control group. We will also include study site fixed effects to account for variation between study sites in HIV retesting. While the inclusion of site fixed effects can reduce power by consuming degrees of freedom, it can also increase precision when there is substantial variation in retesting rates across sites. Given the number of intervention groups, we will account for multiple hypothesis testing by using the Benjamini and Hochberg Procedure to control the false discovery rate [41].

For secondary analyses, we will determine the effect of the interventions relative to control on PrEP uptake (i.e., acceptance of referral to PrEP) at time of retesting HIV-negative by estimating a logistic regression model where the dependent outcome is PrEP uptake, with the model including fixed effects for study community and adjusting for multiple comparisons as described for the primary outcome. We will conduct similar analyses with logistic regression models for the dependent outcomes of HIV seroconversion among participants who retest and linkage to HIV care and ART initiation among those who test HIV-positive. We will report the proportion of participants who retest HIV antibody-positive by study arm and overall, as well as the proportion who link to HIV care and start ART among persons who seroconvert. Although we will be underpowered to detect differences between study arms in HIV-positivity and linkage to care, we will be able to describe these important outcomes among a large sample of adults at risk of HIV exposure and we will identify baseline characteristics that are associated with incident HIV infection using logistic regression models.

Following completion of the study, we will use trial data to examine which interventions are most effective in increasing HIV retesting among three pre-specified subgroups of interest (men aged > 25 years, young women aged 15–24 years, and those reporting > 1 sexual partner in the past 3 months at baseline), given their higher risk of HIV exposure. For standard subgroup analyses, we will examine the effects of each intervention among the three pre-specified subgroups. Heterogeneous effects in subgroups will be estimated using logistic regression models that include interaction terms between indicators of intervention group and subgroup. The results of these analyses will enable us to identify which interventions are most effective in each of the three subgroups and to conduct classical tests for heterogeneity. Restricting our analyses to three pre-specified subgroups will preclude the discovery of large but unexpected treatment effect heterogeneity. To address this challenge in ways that do not spuriously identify treatment effect heterogeneity, we will use machine learning (ML) methods [42–48] to flexibly discover heterogeneous treatment effects across a large set of subgroups that are not specified a priori and are defined by a combination of individual and community characteristics. We will further estimate the potential benefit of using ML-based intervention targeting to improve outcomes, relative to one-size-fits-all or simpler (e.g., subgroup-based) targeting strategies [49].

### Monitoring

The co-principal investigators (MPIs: GC and HT) at UCSF and the University of Pennsylvania will be responsible for study oversight, including data quality assurance and regulatory compliance for adverse event reporting. The study team, including the project manager, co-investigators, data managers, local study coordinators, and research assistants, led by the MPIs, will monitor the progress of the study including recruitment, retention, adverse events, and other challenges on weekly study calls.

To ensure the safety of participants and the integrity of the data, study data will be reviewed every 6 months by an independent data safety monitoring board (DSMB). DSMB members are independent of the study and have no financial, scientific, or other conflict of interests with the trial. The DMSB will review all adverse (AE) and serious adverse events (SAE), as well as enrollment and retention data. The responsibilities of the DSMB will include identifying any patterns of adverse events in accumulated AE reports and reviewing fidelity to the intervention. Following each report, the DSMB will make recommendations on the continuation of the study, as well as provide feedback on any specific challenges or questions posed by the study team.

Safety monitoring will be the responsibility of the MPIs, who will review all AE and SAE reports shared by the field team. Adverse events and harms will be identified through participant report at the single follow-up visit, if and when participants return for HIV retesting. Safety reporting formats and timelines for AEs, SAEs, and other incidents or harms will follow guidelines set forth by the UCSF Institutional Review Board (IRB), the local IRBs and Research and Ethics Committees (RECs) in Kenya and Uganda, and the National Institute of Health (NIH), respectively. All AEs will be documented in study logs, along with their relevant reporting status.

### Dissemination plan

The study’s findings will be prepared for presentation to the scientific community at international and regional academic conferences and for publication in peer-reviewed journals. In addition, findings will be shared with study participants in year 5, through communications in local community dissemination meetings in the study sites. Local community dissemination meetings will be advertised through local leadership structures. We will also prepare documents summarizing the findings and their policy implications for representatives of the Governments of Uganda and Kenya including Ministry of Health officials.

## Discussion

In this trial, we will use an innovative, large, multi-arm (“megastudy”) trial design to compare multiple low-cost behavioral interventions, designed in collaboration with local health care worker and community member input, to promote HIV retesting among persons at high risk for HIV following a baseline negative test in Kenya and Uganda. Ensuring that persons at high risk test regularly for HIV addresses an issue of major public health significance, as frequent, regular HIV testing is a key first step in accessing highly efficacious tools to prevent HIV, ensuring early diagnosis after infection, and achieving prompt ART initiation among people with HIV. As countries approach or meet “95–95–95” targets for HIV treatment and viral suppression, optimizing HIV status awareness and reducing time from HIV infection to diagnosis and treatment initiation is crucial for further reducing HIV incidence. By identifying and implementing low-cost, scalable interventions to promote HIV retesting for individuals at elevated risk of infection across high-volume HTS sites in East Africa, we aim to test and compare multiple interventions in a single trial and thus accelerate discovery of effective (and ineffective) interventions for this public health challenge.

This study has several strengths. By using a megastudy design, we will test multiple behavioral interventions at the same time, across the same populations, and for the same outcome, yielding a like-to-like comparison of efficacy to a standard of care control arm. We will also be able to assess comparative advantages between the interventions. The large sample size will allow for the ability to detect small effects of very low-cost, scalable interventions and to conduct analyses of which interventions work best for key populations. Another study strength is our multi-step adaptation approach to ensure that the intervention content and implementation are acceptable to the local contexts in western Kenya and southwestern Uganda. Our use of avatar videos for intervention delivery has the benefit of controlling intervention consistency across a massive study population rather than relying on counselor delivery, which would have natural variability and possible contamination of messaging across study arms by the counselors. Using avatar videos also allows for low-cost, rapid adjustments based on the workshop and pilot testing feedback; intervention scripts and messengers can be easily and quickly revised, rather than filming new promotional videos with actors.

This study also has several limitations and potential risks. Due to the large scale of the megastudy design, our intervention options were limited to those that could be easily delivered, with low cost and low time burden. We had to eliminate several promising interventions that would have required higher staff numbers and time than was feasible for this trial, such as community-based outreach. There were also some intervention ideas that proposed varying the method of message delivery, such as adding additional follow-up phone calls or including an interactive gamification component. However, varying both the content and method of the interventions in different study arms would have introduced intervention heterogeneity (apart from message content), and thus prevented a clear comparison of messaging content across trial arms. We therefore decided to focus on varying the intervention content while using a standardized delivery method across all study intervention arms (a baseline avatar video and two follow-up SMS messages). Finally, real-world testing in clinical settings is subject to secular trends in testing service delivery that can be impacted by international or domestic funding changes, political turmoil (e.g., elections), or other large-scale events (such as the COVID-19 pandemic). If HIV testing at the participating study clinics is unexpectedly low, it may be challenging to enroll our target sample size of 30,000–40,000 participants. If this situation occurs, we will consider either expanding to additional clinics or using an adaptive design to allow for dropping the least promising interventions at an interim timepoint.

We expect that the methods and results of the IBIS megastudy will provide valuable insights not only for those engaged in HIV service delivery, but also for behavioral scientists who study health-related decision-making and to practitioners working in other areas of public health. Our large sample size will allow for the ability to detect small effects of very low-cost interventions, with the potential for population-level effects when implemented at scale. It will also give us the ability to identify specific interventions that work best for key populations at risk, such as interventions best suited for men and young women, who may respond differently to the intervention message content. We expect to publish results from all 11 intervention arms, including those with null results, thereby reducing publication bias in the field. While other megastudies have been implemented in high-income settings, to our knowledge the IBIS megastudy will be the first of its kind in a low- and middle-income country setting. The methodology can be generalized to many other health problems in low- and middle-income countries (e.g., boosting demand for vaccination). As such, this megastudy has the potential to influence how researchers and programs design and test low-cost, scalable interventions to promote health behaviors. 

## Trial status

The current protocol is version 3.1, as of December 2, 2025. The IBIS megastudy trial is registered under protocol number NCT06971367 in clinicaltrials.gov.

Participatory prototyping workshops were conducted with 53 participants in Kenya in December 2024 and with 52 participants in Uganda in February 2025. The pilot trial is registered under protocol number NCT06785103 in ClinicalTrials.gov. Enrollment of pilot participants occurred from March to April 2025 in Kenya and from May to June 2025 in Uganda. A total of 49 pilot participants were enrolled in each country (98 enrolled overall). Pilot follow-up visits were conducted in Kenya from June to July, 2025. Pilot follow-up visits were unable to be conducted in Uganda due to a temporary delay in funding from the National Institutes of Health (NIH) based on issued Notice Number: NOT-OD-25–104, which introduced a significant policy change regarding foreign (non-US) subawards. Recruitment and enrollment of the IBIS megastudy began on December 9, 2025 and is expected to conclude in June, 2027. Completion of megastudy follow-up visits is expected in December 2027.

## Data Availability

The de-identified final trial dataset, protocol, and statistical analysis plan will be made available without restriction through a publicly accessible data repository.
